# Medical Treatment of Diabetes

**Published:** 1904-09

**Authors:** 


### MEDICAL TREATMENT OF DIABETES.*

The pathology of diabetes mellitus has been the most delusive subject of modern medicine. Repeatedly during the past forty years we have seemed to be on the verge of important discoveries relating to it, only to find that our uncertainties, instead of being

*Read by W. H. Thomson, M.D., LL.D., New York, before the Medical Society of the State of New York.—*Boston Med. and Surg. Jour., Feb.* 25, 1904.

dispelled, had simply taken new forms, which in turn have raised other problems to solve. . . .

As to medicines, I would begin with the statement that I but rarely prescribe opium, codeine or any other of the opium derivatives; and I beg leave to take a short time to explain why, in such a constitutional disease as diabetes, I can not regard this drug as of real service, though it has been long and generally recommended in text-books. Nervines, such as opium, belladonna, strychnine, aconite, etc., are agents which affect certain functions of the nervous system. They, therefore, are only functional remedies, and they have no power whatever in therapeutic doses to directly affect structure or nutrition, however long they be prescribed. All that they ever do is temporarily to produce some functional symptoms, but they never affect the texture or the nutrition of tissues or organs, either beneficially or injuriously. Therefore, none of them can touch diabetes itself any more than they can touch typhoid fever; however, some of them may relieve for a time some serious symptoms in both diabetes and in typhoid fever. As a veteran teacher of materia medica, I used to urge that no functional or symptom medicine, such as the nervines, could be expected to modify an organic disease in any way, either for good or evil, the only exception being in the case of colchicum, which can produce organic changes. Alcohol produces organic changes only by virtue of its chemical and not by its nervine properties. One of the most powerful nervines for properties is nicotine. There is a great deal of functionally active nicotine in a cigar. Let any one who has never smoked a cigar try it, and he will soon illustrate what nicotine can do. Now, my father, my grandfather, and my great-grandfather began to take nicotine by smoking tobacco about the same time of life that I did, namely, at twelve years of age, and continued the same to the last year of their lives, which was at the age of eighty-eight, eighty-seven and ninety-two, respectively, while I am well on to my three-score and ten. Can nicotine, therefore, not injure the bodily mechanism? Never, in rational doses, for like all other nervines, in such cases it can neither do permanent good nor permanent harm.

To this class of functional medicines belongs opium and its derivatives; and, therefore, diabetes continues progressive, however continuously or fully opium is prescribed. A functional medicine is one whose whole action can be secured by one dose. Its hundredth dose does not do any more than the first dose does, sometimes not as much, for with many of this class, as with tobacco, the system becomes used to them. A man may for many years relieve an asthmatic attack by stramonium, but it is not his disease—asthma—which is thereby dealt with, but its symptom, bronchial spasm, and his last dose of stramonium does not do more for him than the first dose did taken years before. More efficacious against his asthma itself would be arsenic and potassium iodide, which no more act in one dose than mercury does in syphilis, or iron in anemia. The fundamental difference between functional medicines, which influence only functions, and organic medicines, which change the nutrition of tissues or organs, would be more widely recognized but for the confusion which exists in many minds about the term "function." Because function is deranged whenever organism is deranged, therefore it is supposed that the converse of this statement is equally true, namely, that a derangement of function must always imply a corresponding derangement of organism. A simple illustration will show the difference. The function of an oil lamp is to give light. Derange the mechanism of the lamp by a leak in the globe, or by an injury to the wick-holder, or by the loss of the wick, and the light-giving function of the lamp is correspondingly deranged. But the entire mechanism of the lamp may be perfectly intact, and yet it will give no light because the source of its function, namely, the oil, is changed by mixing it with water. So a steam engine may be perfect in every detail of its mechanism and yet not go because the source of its function, namely, the coal, is lacking. So in the living mechanism, its functions need power therefor, and that power is in the blood. Functional medicines act only on and through the blood, and not on the tissues. This being so, functional medicines can act only temporarily; they have to be renewed just as the oil in the lamp has to be renewed, though the lamp itself remains the same indefinitely. The oil can remedy no injury to the mechanism of the lamp; and likewise every functional medicine is powerless against a truly organic injurious change going on in the living mechanism.

Functional medicines we need constantly in practice to relieve symptoms, or to meet emergencies. They are indispensable for such purposes, but we should always remember that they are powerless against an organic injurious change going on in the living mechanism. All which a functional medicine can do is to deal with a temporary derangement, not in the structure but in the working, that is, in the function, of the body; and this it does immediately in one dose. But no specifically permanent changes should be expected from it. Opium may affect the symptom, sugar in the urine, by temporarily diminishing it, but it has no effect on the disease causing that symptom.

We must look elsewhere in such a constitutional disease as diabetes than to such functional medicines as opium, and I notice that of late, experience is leading many physicians to abandon its use in this disease. For a number of years I have advocated the free use of cod-liver oil in diabetes. I was specially led to do this by its remarkable effect in the case of two brothers whom I treated thirty-two years ago. The first, an active business man, aged thirty-five, came to me with polyuria, thirst, emaciation and progressive loss of strength, for which he had been treated unavailingly for a year with the usual course of diet and drugs. He then took larger doses of cod-liver oil than any patient in my experience, for he said that he never measured it, but took it directly from the bottle as a drink, followed by Vichy water. In another year's time he was cured, and he has remained well ever since. His brother, two years younger, came to me the following year with much the same condition, as far as the great quantity of sugar in the urine was concerned, but he suffered in addition from general bronchitis. He found that he could stomach cod-liver oil as well as his brother, and with equally good results. The younger the patient, the more persistently I urge the taking of this remedy, and I adopt every resource to make its free use possible by the patient, by the administration with it of pepsin and bismuth. So long as the stomach does not rebel, diabetic patients of this class can not take too much cod-liver oil. Whether this oil acts as a substitute for the starchy elements, or whether it spares the proteids from disintegration, or whether it acts as a nerve food, or whether it acts by increasing the red corpuscles, I do not know; but as an empirical fact I can testify that it both diminishes the sugar and the excess of urea eliminated whenever it is taken freely and is well borne. As in all cases where we aim at influencing nutrition by any measure, it must be used both systematically and perseveringly. Obesity contraindicates it, and likewise I should not give it to patients of very sedentary habits; but it is quite useful with many elderly people.

Next to cod-liver oil, I would mention iron. As before remarked, I have long suspected diabetes as largely a muscle disease, and throughout the animal kingdom muscular power is directly proportioned to the intake of oxygen. As iron can act in us only in its capacity of an oxygen carrier, I try to give diabetics all the iron which they can take, along with all the fresh air which they can get. As iron in many forms tends to cause constipation, which itself not infrequently becomes a serious trouble to diabetics, a very serviceable preparation for them is the old-fashioned Hooper's pills, whose formula is: ℞ Ferri sulph. 3 ss; powdered senna, powdered jalap, cream of tartar, powdered ginger, each, gr. xii; ext. gentiani, q. s. M. Div. in pil.

The progress of medicine in our time has added a class of remedies which are coming more and more into daily use, and to which I would apply the term medicinal, in distinction from surgical, antiseptics. They include a varied list of phenol derivatives, among which I would include quinine, for sooner or later we may look for its synthetic production from coal tar, as well as salicylates and the benzoates. In this class of medicinal antiseptics I would also include arsenic.

It is not improbable that one of the properties of this class of medicines is to start the activity of various enzymes, which in particular states cause derangement of metabolic processes in the body, or lead to disintegration of its tissues. We are only beginning to get some insight into the relations which certain internal secretions bear, on the one hand, to the assimilation of food or, on the other, to the neutralization of poisons in the system, or to the disintegration of the tissues themselves. One of the most striking facts in this connection is the singularly small quantity of the secretions themselves which seems to suffice to maintain the important functions which they subserve. Thus the total extirpation of the pancreas in dogs induces a fatal diabetes. This diabetes is ascribed to the removal of the scattered glandular structure in the pancreas, called the islands of Langerhans. But if three-fourths of the pancreas be excised, so that only one-fourth remains, the small remnant of these island cells is yet enough by their secretion to prevent glycosuria. This indicates that this internal secretion is of the nature of an enzyme, which can propagate its action from very small beginnings. The small bodies, called the parathyroids, have recently been proved to be essential to life, owing to their internal secretion neutralizing a virulent poison, generated most probably in the alimentary canal, and which poison causes fatal tetanic convulsions. So excision of three-fourths of the kidneys, leaving only one-fourth of kidney substance, is followed by death, preceded by great polyuria, with such excessive excretion of urea that it seems as if the proteid tissues were undergoing rapid dissolution. This remarkable result is ascribed to the kidneys, having as their function not only to excrete, but also to regulate the production of urea by an internal renal secretion.

However that may be, I have for some time been much interested with cases which have come under my observation of excessive secretion of urea which, in a way, resembles saccharine diabetes. [Recently] I was consulted by a healthy looking young man, aged twenty-three. He seemed to be a typical neurasthenic, for his complaints were that two years ago he had a sudden nervous breakdown, ever since which he could not apply his mind to anything. He has a constant sense of fatigue, is very nervous and dyspeptic, especially intolerant of starchy food, and very insomnic. His pulse was soft and weak. Examination of his urine showed that he passed no less than 51 Gm. of urea a day, while according to his size and on his restricted diet, he should normally pass only 22 Gm. The total solids of the urine amounted to the high figure of 114.37 Gm., or 1,715 gr. There was no albumen, nor casts, nor sugar in the urine by any test, and he had no fever. His family history was decidedly unfavorable in the respect of neuroses, one brother being in an asylum, an elder sister with something like cyclical insanity, and another sister, younger than himself, is abnormally fleshy.

I put him on the same treatment that I would a diabetic, namely, a shellac-covered capsule, containing 5 gr. of sulphocarbolate of soda and 1 gr. of potassium permanganate, to be taken half an hour after meals and at night; and one hour after meals, t. i. d. a powder of 10 gr. of sodium sulphate and 10 gr. of sodium benzoate. On the 16th he came again, remarkably improved in all his symptoms. His urea had fallen from 51 Gm. to 30 Gm., and the total solids had decreased 250 gr. To add now 10 gr. of aspirin to each dose of the sulphocarbolate, or 40 gr. per diem. On January 16 he reported that he was able to do more work than he had done for some years, and that he was now studying to pass a university examination.

I believe that if physicians, instead of being content in their examinations with discovering whether there were abnormal ingredients in the urine of patients, would also uniformly investigate whether the normal ingredients were present in their healthy proportion, more cases of this kind would be found than are generally suspected. What special process led to such abnormal waste in this patient I do not know, any more than what the real processes are which underlie the disease diabetes. The processes of the chemistry of life are altogether too complex and too imperfectly understood as yet for us to make more than doubtful guesses. But as an empirical fact, I am sure that I succeed better in controlling diabetes mellitus, as far as the use of drugs goes, by a persistent and free administration of antiseptics than by any other means, cod-liver oil and iron excepted. In such a disease the therapeutic tests should be sought for only in severe and prolonged cases; and I will beg leave, in illustration, to cite briefly one such history. He was a gentleman, aged forty-two, who after a year of severe mental strain noticed that he was passing water very often, and that his shirt when wet by the urine was stiffened by it. He was put on diet, opium and codeine, by his physician; but as he was steadily losing flesh and strength and was subject to alarming attacks of syncope, he finally consulted me. I first saw him after one of these attacks. He was cyanosed and extremely dyspneic. Another attack soon occurred, in which he seemed to be passing into diabetic coma. For sixteen years after this I conducted the management of his case, watching him very closely, as he was a most intimate friend of mine, and though I never found sugar absent from his urine, during those sixteen years he ably performed the exacting duties of chief editor of one of the largest of New York's daily newspapers. He then moved to another city, to enter upon a much less exacting business. I warned him that he was by no means a well man, and that he should continue his treatment without change. I learned, however, that he became quite careless, and in three months he succumbed to an attack of bronchitis. Such histories of years of active life while keeping up treatment, soon followed by fatal decline on cessation of the same, have occurred more than once in my experience. Nature does not cure diabetes.

As remarked before, I would include arsenic in this class of antiseptics, as it comes nearer in its properties as a medicine to such agents than to any other. My usual practice is to combine it in the same prescription with them, and, therefore, I ordinarily give arsenous acid itself, watching for the development of arsenical symptoms, just as in prescribing it for any other purpose. Some of the liquid preparations, as Fowler's solution, the liq. arsenici hydrochlorici or the bromide of arsenic, have the advantage that the dose may be increased or diminished more readily; but I have failed to note any special advantage in changing the form of the drug, because it is arsenic itself in them all to which the effect is due. As to the rest, their administration should be varied from time to time, on the general principle that the too continued use of any drug tends to lessen its efficacy, or in the case of the salicylates to irritation of the kidneys. If called to a patient who is voiding so large an amount of sugar that a speedy reduction of it is imperative, I would give 15 gr. of antipyrine, with the same of sod. benzoate, four times a day. After a time I would substitute 15 gr. of aspirin with 10 of bismuth salicylate. In subacute cases, illustrative prescriptions would be somewhat as follows: Benzosol, gr. 48; sodium benzoate, dr. 4; arsenous acid, gr. 1; sodium salicylate, dr. 11. Make 48 capsules. Two an hour after meals and at night. Or, sodium sulphocarbolate, dr. 2; salicin, dr. 1; phenacetin, dr. 2; ammonia benzoate, dr. 4. Make 48 capsules, shellac covered. Two a half hour after meals; and if there be much insomnia, 20 gr. of strontium bromide and 15 of antipyrine at night. Of course, each physician can vary such medication according to indications. A weekly dose of blue pill is nearly always beneficial to patients after middle life. Unless the patient be much emaciated, one of the means which works well to prevent the tendency to constipation is a powder, dissolved in a tumbler of hot water and sipped slowly on rising, of a dram and a half of sod. bicarb., half a dram each of sod. sulphate and of magnes. sulphate, and 10 gr. of sod. salicylate.

Alkalies, in the form of mineral waters, have always held a deserved place in the treatment of diabetes. There is, however, one caution to be always borne in mind, and that is that the free use of all saline water tends to increase the waste of the system, so to speak, and they should not be given to any one who is really losing flesh. I think that I have seen them do harm to emaciating diabetics.

Diabetes is a very serious disease on one account alone, if not on other accounts, in that it virtually compels a starvation of an essential element of food. The system must have sugar, and if it does not get the sugar which it can use in the food, it will turn to its own proteid for sugar. Along with this profound perversion poisons begin to be generated, of which oxybutyric acid and its derivatives are doubtless only a part, and which poisoning we are practically unable to neutralize. Our function, therefore, is to put off the evil day of these terminal developments as long as possible and to do that, I am sure that merely functional medicines will afford us no help. The essentials of the chemistry of this specific toxemia are yet unknown. The formation of acetone and of diacetic acid occurs early, but the blood itself often changes its color, owing to the abundant presence in it of fatty and of proteid granules. Something, however, holds back the carbon dioxide in the tissues, for it is a mistake to ascribe the low percentage of carbonic acid in the blood to its hyperacidity, because the blood of these patients when drawn will take up oxygen or carbonic acid about as freely as normal blood. The last step is the rapid disappearance of sugar from the urine. Why sugar is absent in diabetic coma we do not know, but significantly enough the first danger signal comes from the kidneys, for before drowsiness begins the urine often deposits an enormous number of peculiar looking casts. To postpone the well-nigh inevitable death in these conditions, I rely upon the most trustworthy diuretic which we possess, namely, prolonged intestinal irrigation with hot normal saline solution, by means of the best instrument for the purpose which I know of, Kemp's rectal irrigator. Hypodermic injections of 7 to 14 gr. of camphor in sterilized almond or olive oil answer best to sustain the failing heart, while diluted milk holding all the sodium bicarbonate which the patient can take is the only recourse left for food. Some of the fatal poison doubtless could be washed out if we could bleed the patient freely and transfuse an equivalent amount of normal saline.

